# Quercetin Reduces *Toxoplasma gondii* Infection in In Vitro and Ex Vivo Placental Models

**DOI:** 10.3390/ijms27136054

**Published:** 2026-07-06

**Authors:** Muriel Pereira Souto, Guilherme Vieira de Faria, Guilherme de Souza, Joed Pires de Lima Júnior, Izadora Santos Damasceno, Marcos Paulo Oliveira Almeida, Natalia Carine Lima dos Santos, Rafael Martins de Oliveira, Emanuelle Lorrayne Ferreira, Luana Carvalho Luz, Tarcísio Paiva Mendonça, Cecília Silva Pereira, Foued Salmen Espindola, Allisson Benatti Justino, Anna Laura de Jesus Gomes, Rosiane Nascimento Alves, Thales A. M. Fernandes, Eloisa Amália Vieira Ferro, Bellisa Freitas Barbosa, Samuel Cota Teixeira

**Affiliations:** 1 Laboratory of Immunophysiology of Reproduction, Institute of Biomedical Science, Universidade Federal de Uberlândia, Uberlândia 38405-317, Brazil; muriel.souto@ufu.br (M.P.S.); guilherme.vieira@ufu.br (G.V.d.F.); guilhermesouza@ufu.br (G.d.S.); joed.lima@ufu.br (J.P.d.L.J.); izadora.damasceno@ufu.br (I.S.D.); marcospaulooliveiraalmeida@ufu.br (M.P.O.A.); carine.natalia@ufu.br (N.C.L.d.S.); rafael_martinso@ufu.br (R.M.d.O.); emanuelle.ferreira@ufu.br (E.L.F.); luanaclz@ufu.br (L.C.L.); eloisa.ferro@ufu.br (E.A.V.F.); bellisafb@ufu.br (B.F.B.); 2 Laboratory of Biochemistry and Molecular Biology, Institute of Biotechnology, Universidade Federal de Uberlândia, Uberlândia 38405-317, Brazil; tarcisio.paiva.mendonca@ufu.br (T.P.M.); cecilia.pereira@ufu.br (C.S.P.); foued@ufu.br (F.S.E.); allisonbjustino@ufu.br (A.B.J.); 3 Laboratory of Immunopathology, Institute of Biomedical Sciences, Universidade Federal de Uberlândia, Uberlândia 38405-317, Brazil; anna.gomes@ufu.br; 4 Department of Agricultural and Natural Science, Universidade do Estado de Minas Gerais, Ituiutaba 38302-192, Brazil; rosiane.alves@uemg.br; 5 Department of clinical medicine, Medicine College, Universidade Federal de Uberlândia, Uberlândia 38405-317, Brazil; 6 Laboratory of Applied Toxinology, Butantan Institute, São Paulo 05503-900, Brazil; t.fernandes.proppg@proppg.butantan.gov.br

**Keywords:** alternative treatment, natural products, quercetin, congenital toxoplasmosis, maternal–fetal interface, placenta

## Abstract

Congenital toxoplasmosis, caused by the obligatory intracellular apicomplexan protozoan *Toxoplasma gondii*, can lead to severe complications during pregnancy, including fetal malformations and spontaneous abortion. In the present study, the anti-*T. gondii* effects of the natural flavonoid quercetin were evaluated using in vitro, ex vivo, and in silico models. Cell viability and intracellular proliferation of the parasite were determined via colorimetric assays. The lipid droplet assay was analyzed using Nile Red staining, the antioxidant and oxidative stress parameters were determined by biochemical assays, and the cytokine levels were quantified by immunoassays. Our results demonstrated that non-cytotoxic concentrations of quercetin (CC_50_ > 100 μM) significantly inhibited parasite proliferation (IC_50_ = 14.10 ± 2.83 μM; SI > 7.09) in an irreversible manner. Quercetin impairs parasite adhesion, invasion, and reinfection capacity. In parallel, quercetin reduced lipid droplet accumulation, restored antioxidant balance by modulating redox biomarkers, and regulated cytokine production, notably increasing IL-4, IL-6, and IL-8 levels. Corroborating the in vitro findings, quercetin significantly reduced *T. gondii* proliferation in human placental villous explants while preserving tissue architecture and viability. In silico analyses revealed that quercetin binds to the active site of *T. gondii* hypoxanthine-guanine phosphoribosyltransferase (TgHGPRT) and exhibits favorable pharmacokinetic and drug-likeness properties.

## 1. Introduction

Toxoplasmosis is a disease caused by the apicomplexan obligate intracellular protozoan *Toxoplasma gondii*, capable of accidentally infecting humans. The infection is transmitted by the fecal–oral route and requires special attention due to the risk of congenital infection [1,2]. In Brazil, the congenital infection with this parasite is highly prevalent and represents a public health problem with a prevalence of 3.4 cases per 1000 live births [3,4,5].

The placenta acts as a protective barrier against pathogens such as *T. gondii*, while also mediating the exchange of nutrients, gases, and waste between the mother and fetus [6]. Maternal infection during or shortly before pregnancy may lead to congenital toxoplasmosis, with outcomes ranging from miscarriage or neonatal death to postnatal manifestations such as ventriculomegaly, intracranial calcifications, and chorioretinitis [7]. Moreover, the risk of vertical transmission increases almost linearly as gestational age progresses, rising from approximately 6% at 13 weeks to 40% at 26 weeks and reaching 72% at 36 weeks of gestation [8].

However, the success of *T. gondii* infection depends, among other mechanisms, on the host’s immune response. Although the functions of CD4+ T cell subpopulations are not well understood, including Th1, Th2, Th17, and regulatory T cells, the Th1 response is essential for intracellular pathogens such as *T. gondii* [9]. However, at the maternal–fetal interface, a balanced immune environment is necessary, as excessive inflammation can be harmful to the host [10].

Once maternal infection is confirmed during prenatal care, spiramycin is prescribed for pregnant women under 18 weeks of gestation with no confirmed fetal infection. For those at 18 weeks of gestation or more with a diagnosis of fetal infection, the combination of pyrimethamine and sulfadiazine (SP) is recommended [11]. However, conventional treatments for *T. gondii* present significant challenges due to their adverse effects. Pyrimethamine is associated with neutropenia and can trigger hepatotoxicity, aplastic anemia, leukopenia, dose-dependent bone marrow suppression, thrombocytopenia, and hypersensitivity reactions [12]. Sulfadiazine, on the other hand, can lead to allergic dermatitis, urticaria, and neutropenia [12,13,14]. Furthermore, the development of SP-resistant strains has been reported, further limiting the effectiveness of current therapeutic regimens [15,16].

Thus, the cumulative adverse effects on the mother–child dyad, combined with the emergence of drug-resistant strains, highlight the urgent need to search for alternative treatments against congenital toxoplasmosis. In this context, natural products have been studied due to their pharmacotherapeutic potential [17]. Quercetin is the most abundant non-toxic natural flavonoid in the human diet, commonly present in fruits and vegetables, and exhibits anti-inflammatory, anti-apoptotic, and antioxidant properties [18]. Importantly, quercetin is known to modulate oxidative stress and cellular defense pathways, which are critical factors in host–parasite interactions and in the stress-induced differentiation of *T. gondii* [19,20]. It has also been shown to reduce adipocytes [21], as host lipids are commonly associated with the pathogenesis of *T. gondii* [22]. Moreover, this bioactive compound has demonstrated antiparasitic properties against *Leishmania* spp., *Trypanosoma* spp., *Plasmodium* spp., *Cryptosporidium* spp., *Trichomonas* spp., and *T. gondii* [23].

To date, there are no studies on the action of quercetin in *T. gondii* infection in models of the maternal–fetal interface. Therefore, two study models were used: an in vitro model with human BeWo trophoblastic cells and an ex vivo model with third-trimester human chorionic villi. In silico analyses were also conducted to evaluate the potential targets of quercetin and its pharmacological properties. The results indicate that quercetin may constitute a safer and more effective therapeutic alternative, addressing an important public health challenge.

## 2. Results

### 2.1. Quercetin Controls the T. gondii Proliferation, Prevents Reinfection of New Cells, and Maintains Cell Viability

**Figure 1:**
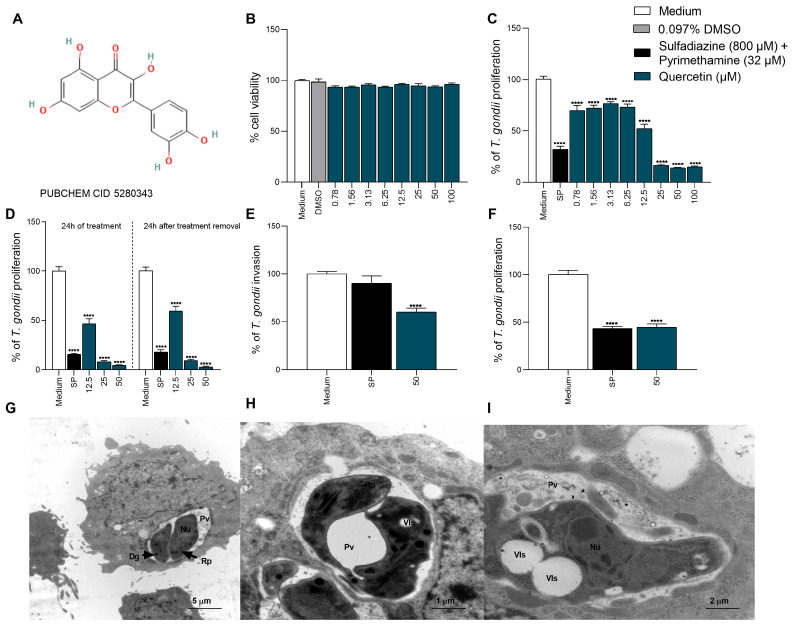
Quercetin irreversibly reduces *T. gondii* intracellular proliferation and prevents parasites from reinfecting new cells. (**A**) Chemical structure of quercetin (C_15_H_10_O_7_). (**B**) Viability of BeWo cells exposed to quercetin treatment for 24 h at concentrations in a two-fold serial dilution (ranging from 0.78 to 100 μM), a combination of sulfadiazine (800 μM) and pyrimethamine (32 μM) (SP), or culture medium alone, considered as 100% cell viability. The percentage of viability was analyzed using the colorimetric MTT method previously described. (**C**) *T. gondii* proliferation in BeWo cells was analyzed by the β-galactosidase colorimetric assay. BeWo cells were infected and subsequently treated with quercetin in a two-fold serial dilution (ranging from 0.78 to 100 μM), a combination of sulfadiazine (800 μM) and pyrimethamine (32 μM) (SP), or culture medium alone, considered as 100% parasite proliferation. (**D**) BeWo cells were treated for 24 h with quercetin (12.5, 25, and 50 μM), SP, or culture medium alone after the *T. gondii* infection. In addition, another microplate with the same treatments was performed, differing in that the treatment was removed from the infected cells; thus, the cells were maintained for an additional 24 h with culture medium only. All *T. gondii* proliferation assays were performed using the β-galactosidase colorimetric assay. This assay assesses the reversibility of the treatment. (**E**,**F**) BeWo cells were infected for 24 h and treated with quercetin at 50 μM, SP, or culture medium alone. After 24 h, the treated parasites were removed from within the cells and, consequently, from the parasitophorous vacuoles, and used to reinfect new BeWo cells. Invasion (**E**) and proliferation (**F**) capacity under reinfection conditions were analyzed. (**G**–**I**) Electron micrographs of *T. gondii* after a 24 h exposure to (**G**) culture media alone (control group), (**H**) SP (800 μM and 32 μM) and (**I**) quercetin (50 μM). Nu stands for nucleus; Vls for vesicle-like structure; Dg for dense granule of *T. gondii* tachyzoites; and Pv for parasitophorous vacuole. Scale bar: 2 µm (located in the lower right corner). Mean ± standard error of the mean (SEM) was used for statistical analyses in this study. * Indicates comparison of the control (medium) with the treatments. The tests applied were one-way analysis of variance (ANOVA), followed by Dunnett’s post hoc test for multiple comparisons. Statistically significant differences were considered when *p* < 0.05.

First, the cytotoxicity of quercetin (Figure 1B) was evaluated in cells treated with a twofold serial dilution at concentrations ranging from 0.78 to 100 μM for 24 h. None of the tested concentrations caused a loss of viability compared with the untreated control (medium), resulting in a CC50 > 100 μM. Next, the ability of quercetin to inhibit parasitism in *T. gondii*–infected BeWo cells was also evaluated. All quercetin concentrations significantly reduced parasitism compared with the untreated control group (**** *p* < 0.0001; Figure 1C). As expected, the classical drug combination of sulfadiazine + pyrimethamine (SP) also controlled the intracellular parasite proliferation compared to the control group (**** *p* < 0.0001; Figure 1C). The IC50 value of quercetin was 14.10 ± 2.83 μM, and the selectivity index (SI) was > 7.09. Subsequently, a reversibility assay was performed to assess whether quercetin sustains its potent anti-*T. gondii* effect for more than 24 h, even in the absence of treatment. Quercetin (12.5, 25, and 50 μM; **** *p* < 0.0001) and SP (**** *p* < 0.0001) significantly controlled parasite proliferation after 24 h of treatment, and their effects were extended for an additional 24 h compared with the untreated control (medium), even after removal of the treatments from *T. gondii*–infected BeWo cells (Figure 1D). Furthermore, a reinfection assay was conducted to analyze the ability of quercetin-treated intracellular parasites to reinfect new host cells. Our data demonstrated that quercetin (50 μM) impaired the invasion and subsequent intracellular proliferation of parasites obtained from infected/treated BeWo cells compared to the control group (**** *p* < 0.0001; Figure 1E,F). In contrast, intracellular tachyzoites collected from SP-infected/treated BeWo cells showed impairment only in parasite proliferation (**** *p* < 0.0001; Figure 1F). Finally, we conducted an ultrastructural analysis of intracellular tachyzoites of *T. gondii* treated with quercetin to investigate the direct effect of the drug on the morphology of the parasite. The BeWo cells were infected and treated with only culture medium and quercetin (50 µM) for 24 h. Next, the infected cells were fixed and processed for transmission electron microscopy (TEM). Untreated cells harbored a parasitophorous vacuole (Pv) containing tachyzoites with a characteristic arc shape, a well-defined tubulovesicular structure, rhoptries (Rp), nucleus (Nu), and dense granule (Dg), which suggested a normal endogenous replication process (Figure 1G). As described previously [24], SP treatment induced the formation of intracellular vacuole-like structures (Vls) and parasites joined by their basal ends (Figure 1H). Additionally, quercetin promoted a notable change in the morphology of the parasite. Infected cells treated with quercetin frequently showed intracellular vacuole-like structures (Vls) (Figure 1I).

### 2.2. Quercetin Impairs Early Steps of T. gondii Infection by Directly Targeting Both the Host Cell and the Parasite

**Figure 2:**
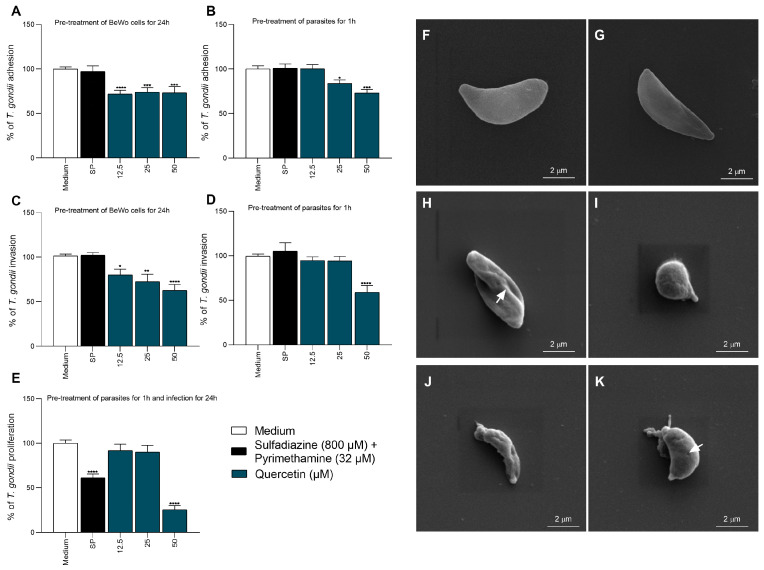
Quercetin inhibits the adhesion and invasion of *T. gondii* in BeWo cells and induces structural damage to the parasite. (**A**,**B**) We performed pre-treatment of host cells for 24 h (**A**) and pre-treatment of the parasite for 1 h (**B**) with quercetin (12.5, 25, and 50 μM), SP, or culture medium alone. The ability of the parasite to adhere to previously fixed cells was then analyzed. The percentage of *T. gondii* adhesion was measured using the β-galactosidase assay. (**C**) Shows the invasion results of cells pre-treated for 24 h with quercetin (12.5, 25, and 50 μM), SP, or culture medium alone. For this assay, only parasites that were able to enter the cells after 3 h of infection were quantified using the β-galactosidase assay. (**D**) Parasites were pre-treated for 1 h under quercetin (12.5, 25, and 50 μM), SP, or culture medium alone conditions. After 3 h of incubation, the ability of quercetin-pre-treated parasites to invade host cells was evaluated, also using the β-galactosidase assay. (**E**) Parasites were pre-treated for 1 h under quercetin (12.5, 25, and 50 μM), SP, or culture medium alone conditions. After 24 h, the β-galactosidase assay was performed to analyze the percentage of parasites that proliferated following 1 h parasite pre-treatment. (**F**) Parasites pre-treated for 1 h with culture medium alone and analyzed by scanning electron microscopy. (**G**) Parasites pre-treated for 1 h with the SP combination (800 and 32 μM, respectively) and analyzed by scanning electron microscopy. (**H**–**K**) Parasites pre-treated for 1 h with quercetin at 50 μM and analyzed by scanning electron microscopy. Scale bars are shown in the lower right corner (2 μm). White arrows indicate structural alterations. Mean ± standard error of the mean (SEM) was used for statistical analyses in this study. * Indicates comparison of the control (medium) with the treatments. The tests applied were one-way analysis of variance (ANOVA), followed by Dunnett’s post hoc test for multiple comparisons. Statistically significant differences were considered when *p* < 0.05.

To identify the possible targets of quercetin, we pre-treated *T. gondii* tachyzoites (1 h) and BeWo cells (24 h) and assessed both the adhesion and invasion rate by β-galactosidase activity. To evaluate the effects of pre-treatment of either host cells or parasites on *T. gondii* infection dynamics, several functional assays were performed using only culture medium, SP (800 µM and 32 µM, respectively), and the selected concentrations of quercetin (12.5, 25, and 50 μM). Initially, parasite adhesion to host cells was assessed after pre-treatment of BeWo cells or parasites, both evaluated using the β-galactosidase assay. In this context, quercetin reduced parasite adhesion at all tested concentrations when host cells were pre-treated compared with untreated control cells (12.5 **** *p* < 0.0001, 25 *** *p* = 0.0002, and 50 μM *** *p* = 0.0001; Figure 2A). Similarly, pre-treatment of parasites with 25 and 50 μM of quercetin reduced adhesion compared to untreated parasites (* *p* = 0.0241, *** *p* = 0.0001; Figure 2B). In contrast, SP treatment did not significantly alter parasite adhesion under either experimental condition (Figure 2A,B). Subsequently, the effects of pre-treatment on parasite invasion and intracellular development were evaluated. When host cells were pre-treated for 24 h prior to infection, quercetin reduced parasite invasion at 12.5, 25, and 50 μM compared with untreated control cells (* *p* = 0.0360, ** *p* = 0.0033, **** *p* < 0.0001; Figure 2C). Similarly, when parasites were pre-treated for 1 h prior to infection, quercetin reduced invasion at 50 μM compared with untreated parasites (**** *p* < 0.0001; Figure 2D), while SP treatment did not significantly affect parasite invasion in these conditions (Figure 2C,D). Furthermore, the proliferative capacity of parasites was evaluated at 24 h after infection using parasites pre-treated for 1 h. In this assay, SP significantly reduced parasite proliferation compared with untreated control (**** *p* < 0.0001; Figure 2E). In parallel, quercetin markedly reduced parasite proliferation at 50 μM compared with untreated parasites (**** *p* < 0.0001; Figure 2E), whereas lower concentrations (12.5 and 25 μM) did not significantly affect parasite proliferation. Given that quercetin pre-treatment impaired early steps of infection, parasite morphology was investigated by scanning electron microscopy (SEM). Our data demonstrated that parasites treated with culture medium or SP for 1 h exhibited a typical crescent shape featuring a smooth, regular surface and an evident conoid (Figure 2F,G). In contrast, quercetin-pre-treated parasites presented distortions, irregular rough surface, papules and dimples (white arrows) (Figure 2H–K). Based on these findings, quercetin at 50 μM was selected for all subsequent assays, as this concentration was demonstrated to be safe and effective, consistently exhibiting strong anti-*T. gondii* activity.

### 2.3. Quercetin Downregulates Lipid Droplet (LD) Production During T. gondii Infection

**Figure 3:**
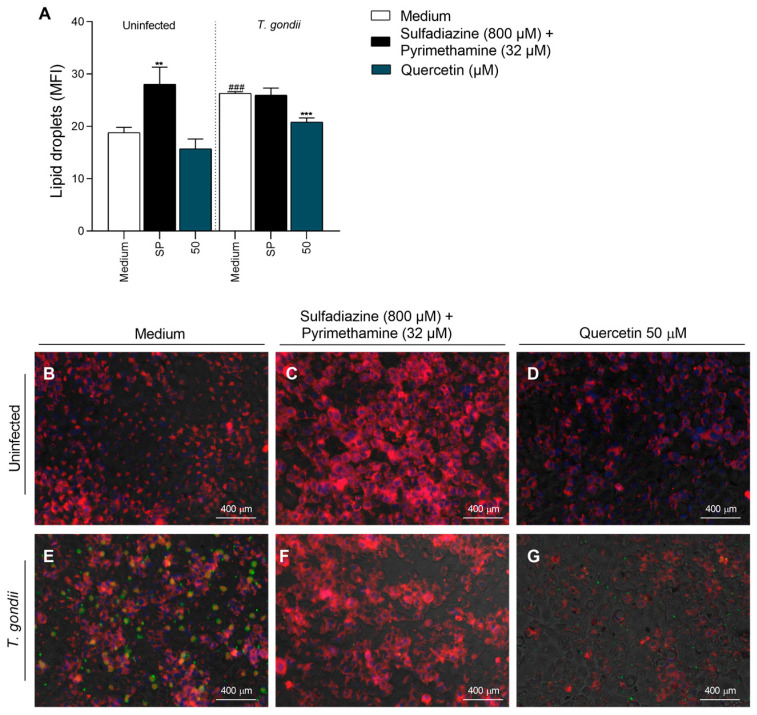
Quercetin modulates lipid droplet formation in BeWo cells infected with *T. gondii*. (**A**) Shows the results of LD formation in cells infected or not with *T. gondii* and treated with quercetin (50 μM), SP, or culture medium for 24 h, stained with Nile Red. Representative images illustrate the distribution of LDs where cell nucleus is blue, LDs are red, and *T. gondii* tachyzoites are green. Thus, it is possible to visualize the phenomenon according to each condition: (**B**) non-infected and untreated cells, (**C**) non-infected cells treated with SP, (**D**) non-infected cells treated with quercetin, (**E**) infected and untreated cells, (**F**) infected cells treated with SP, and (**G**) infected cells treated with quercetin. Scale bars are shown in the lower right corner (400 μm). Mean ± standard error of the mean (SEM) was used for statistical analyses in this study. * Indicates comparison of the control (medium) with the treatments. ^#^ Indicates comparison between control groups (medium) in non-infected and infected conditions. The tests applied were one-way analysis of variance (ANOVA), followed by Dunnett’s post hoc test for multiple comparisons. Statistically significant differences were considered when *p* < 0.05.

To elucidate the mechanisms of action of quercetin against *T. gondii* in BeWo cells, we assessed its effect on lipid droplets (LDs) biogenesis. Our results demonstrated that quercetin (50 μM) did not modulate LD production in uninfected BeWo cells compared to uninfected/untreated cells. In contrast, SP treatment increased LD levels in uninfected cells compared to the control group (** *p* = 0.0071; Figure 3A). Moreover, *T. gondii* infection itself upregulated LDs production in relation to uninfected/untread cells (^###^ *p* = 0.0004; Figure 3A). Interestingly, during *T. gondii* infection, in which quercetin-treated cells showed a significant downregulation of the LD biogenesis compared to untreated cells (medium) (*** *p* = 0.0002; Figure 3A). The fluorescence microscopy reinforces these findings (Figure 3B–G). Uninfected/untreated cells (Figure 3B) showed less stain for LDs than infected/untreated cells (Figure 3E), and SP treatment increased LD labeling (Figure 3C,F). In the absence of infection, quercetin demonstrated a similar pattern of LDs distribution like that of uninfected/untreated (Figure 3D). However, it caused a prominent decrease in LDs levels in *T. gondii*-infected cells (Figure 3G).

### 2.4. Quercetin Modulates Oxidative Stress and Antioxidant Biomarkers

**Figure 4:**
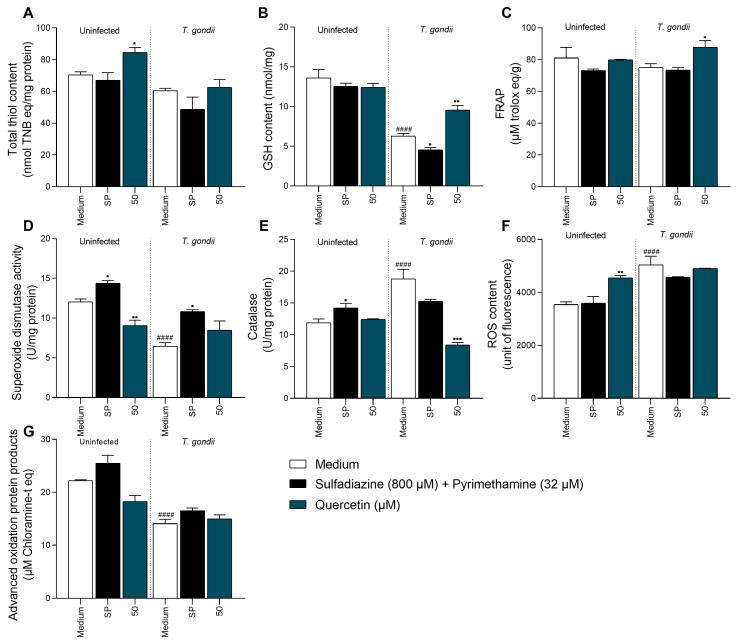
Quercetin exhibits antioxidant activity in BeWo cells. BeWo cells were infected or not with *T. gondii* and treated with quercetin (50 μM), SP, or culture medium alone. Analyses were performed for (**A**) total thiol content, (**B**) reduced glutathione (GSH) content, (**C**) total antioxidant capacity (FRAP), (**D**) superoxide dismutase (SOD) activity, (**E**) catalase (CAT) activity, (**F**) ROS content, and (**G**) advanced oxidation protein products (AOPP). Mean ± standard error of the mean (SEM) was used for statistical analyses in this study. * Indicates comparison of the control (medium) with the treatments. ^#^ Indicates comparison between control groups (medium) under non-infected and infected conditions. The tests applied were one-way analysis of variance (ANOVA), followed by Dunnett’s post hoc test for multiple comparisons. Statistically significant differences were considered when *p* < 0.05.

To decipher the antiparasitic action of quercetin, we performed biochemical assays of antioxidant defenses and damage markers. Non-infected and infected cells were treated with SP, quercetin (50 μM), or culture medium alone (medium). Quercetin significantly increased total thiol content compared to the control group under uninfected conditions (* *p* = 0.0444; Figure 4A). Regarding GSH levels, *T. gondii* infection reduced the levels of this antioxidant in relation to the uninfected/untreated group (^####^ *p* < 0.0001). Under parasite infection, SP reduced GSH levels, whereas quercetin increased GSH production, when compared to the infected/untreated cells (* *p* = 0.0477, ** *p* = 0.0026, respectively; Figure 4B). Concerning total antioxidant capacity, quercetin increased FRAP in infected cells compared to the infected/untreated cells (* *p* = 0.0413; Figure 4C).

Moreover, *T. gondii* infection reduced SOD activity compared to uninfected/untreated cells (^####^ *p* < 0.0001; Figure 4D). Under non-infected conditions, SP increased SOD levels, while quercetin reduced SOD levels compared to non-infected/non-treated cells (* *p* = 0.0248; ** *p* = 0.0088, respectively). During the infection, SP increased SOD levels compared to the infected/untreated cells (* *p* = 0.0100), while quercetin did not show a statistically significant difference. Regarding catalase activity, the infection increased enzyme activity compared to the uninfected/non-treated cells (^####^ *p* < 0.0001; Figure 4E). Under non-infected conditions, SP increased catalase activity (* *p* = 0.0417), while under infected conditions, quercetin reduced catalase activity compared to infected/untreated cells (*** *p* = 0.0004).

Finally, oxidative damage and stress markers were evaluated through the production of ROS and AOPP. The infection increased ROS levels compared to non-infected/non-treated cells (^####^ *p* < 0.0001; Figure 4F), and quercetin increased ROS production under non-infected conditions (** *p* = 0.0092). Under infection, no statistically significant differences were observed between the treatments. Moreover, the isolated infection reduced AOPP levels compared to non-infected/non-treated cells (^####^ *p* < 0.0001; Figure 4G), with no differences observed between the treatments within each experimental condition.

### 2.5. Quercetin Significantly Upregulates IL-4, IL-6, and IL-8 Levels in BeWo Cells

**Figure 5:**
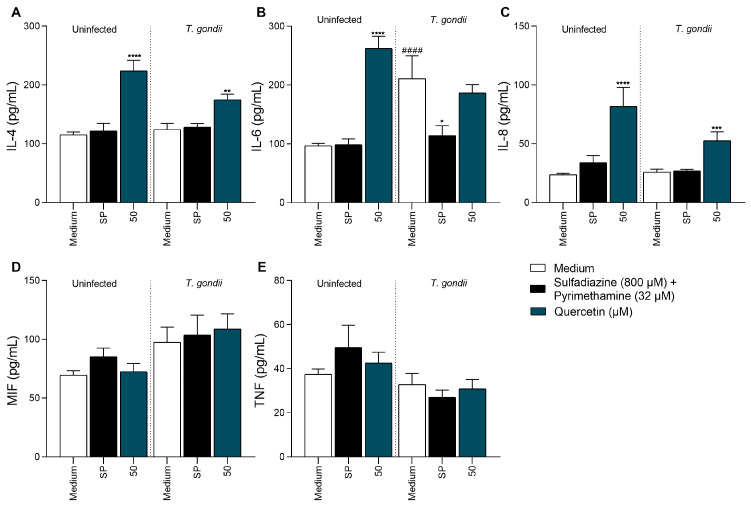
Quercetin increases IL-4 and IL-8 production in *T. gondii*-infected cells. BeWo cells were infected or not with *T. gondii* and treated with quercetin (50 μM), SP, or culture medium alone. Cell supernatants were measured for (**A**) IL-4, (**B**) IL-6, (**C**) IL-8, (**D**) MIF, and (**E**) TNF. Mean ± standard error of the mean (SEM) was used for statistical analyses in this study. * Indicates comparison of the control (medium) with the treatments. ^#^ Indicates comparison between control groups (medium) under non-infected and infected conditions. The tests applied were one-way analysis of variance (ANOVA), followed by Dunnett’s post hoc test for multiple comparisons. Statistically significant differences were considered when *p* < 0.05.

To evaluate the impact of quercetin on the modulation of the host’s immune response, we measured the levels of the cytokines IL-4, IL-6, IL-8, MIF, and TNF-α secreted by BeWo cells under different experimental conditions. Regarding IL-4 production, quercetin treatment increased cytokine levels in non-infected cells compared to non-infected/untreated cells (**** *p* < 0.0001; Figure 5A). Similarly, under infection, quercetin also increased IL-4 levels compared to infected/untreated cells (** *p* = 0.0017; Figure 5A). Regarding IL-6, *T. gondii* infection increased cytokine levels compared to non-infected/non-treated cells (^####^ *p* < 0.0001; Figure 5B). In infected conditions, treatment with SP reduced IL-6 levels compared to infected/untreated cells (* *p* = 0.0258; Figure 5B). In contrast, treatment with quercetin increased IL-6 production compared to the uninfected/untreated cells (**** *p* < 0.0001; Figure 5B). For IL-8 production, treatment with quercetin increased cytokine levels in uninfected cells compared to uninfected/untreated cells (**** *p* < 0.0001; Figure 5C). Similarly, during the infection, quercetin increased IL-8 levels compared to infected/untreated cells (*** *p* = 0.0009; Figure 5C). No statistically significant differences were observed in MIF and TNF-α levels between the infected and uninfected groups or between the treatments (Figure 5D,E).

### 2.6. Quercetin Reduces T. gondii Proliferation While Preserving Placental Tissue Viability

**Figure 6:**
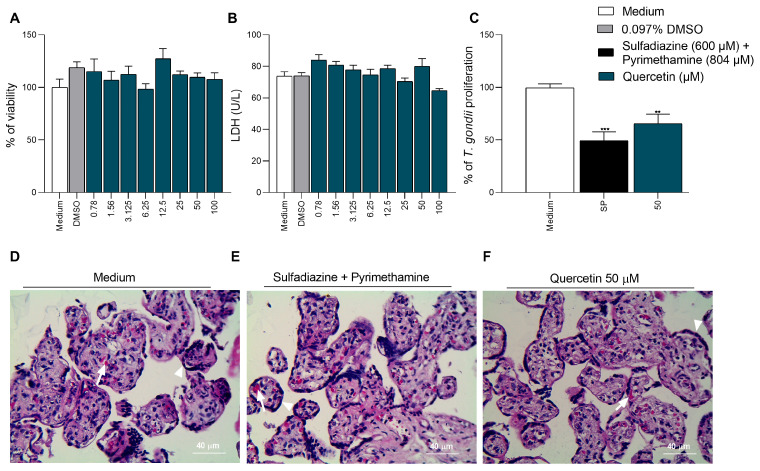
Quercetin inhibits *T. gondii* proliferation in human placental villi and maintains tissue viability. (**A**) Tissue viability was analyzed by MTT and (**B**) LDH assays over a concentration range of 0.78 to 200 μM. (**C**) Infected villous explants were treated with quercetin (50 μM), SP, or culture medium alone for 24 h. Parasite proliferation was measured by the β-galactosidase colorimetric assay. Tissue viability was also assessed by histological sections stained with hematoxylin and eosin (H&E), as shown in the photomicrographs: (**D**) culture medium alone, (**E**) SP treatment, and (**F**) quercetin at 50 μM. Scale bars are shown in the lower right corner (40 μm). Mean ± standard error of the mean (SEM) was used for statistical analyses in this study. * Indicates comparison of the control (medium) with the treatments. White arrows indicate fetal blood vessels. Arrowheads indicate the outer layer of multinucleated syncytiotrophoblast. The tests applied were one-way analysis of variance (ANOVA), followed by Dunnett’s post hoc test for multiple comparisons. Statistically significant differences were considered when *p* < 0.05.

Finally, to support the in vitro findings, we performed assays using human villous explants as ex vivo experimental models. Initially, the viability of placental tissues exposed to quercetin was evaluated. Based on the MTT assay, the explants treated with different concentrations of quercetin did not show reductions in viability (Figure 6A). Similarly, the analysis of LDH release demonstrated that none of the tested concentrations induced cytotoxicity, showing LDH levels comparable to untreated explants (Figure 6B). After confirming that quercetin was not toxic to placental explants, we evaluated its effect on the *T. gondii* proliferation. As expected, treatment with SP significantly reduced the proliferation of the parasite compared to untreated infected explants (*** *p* = 0.0004; Figure 6C). Similarly, quercetin at 50 μM also reduced parasite proliferation compared to untreated infected explants (** *p* = 0.0091; Figure 6C). To complement the biochemical findings, we morphologically assessed the villous integrity using hematoxylin and eosin staining. Histological analysis demonstrated that untreated explants (Figure 6D), as well as those treated with SP (Figure 6E) and quercetin (Figure 6F), exhibited a preserved villous architecture, with well-defined structures, intact syncytiotrophoblast layer, and preserved mesenchymal nucleus, without visible structural alterations under any of the treatments.

### 2.7. Molecular Docking Reveals Quercetin Binding to TgHGPRT and ADMET Properties

**Figure 7:**
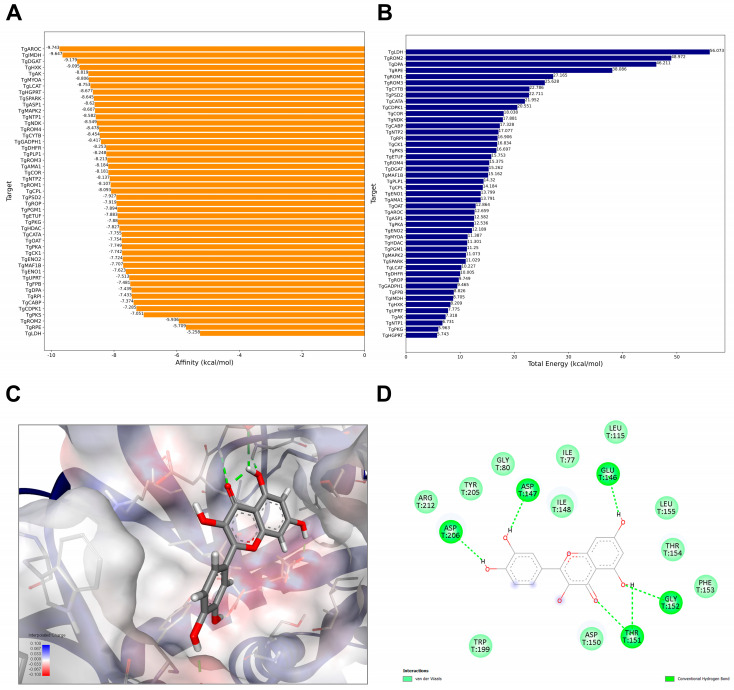

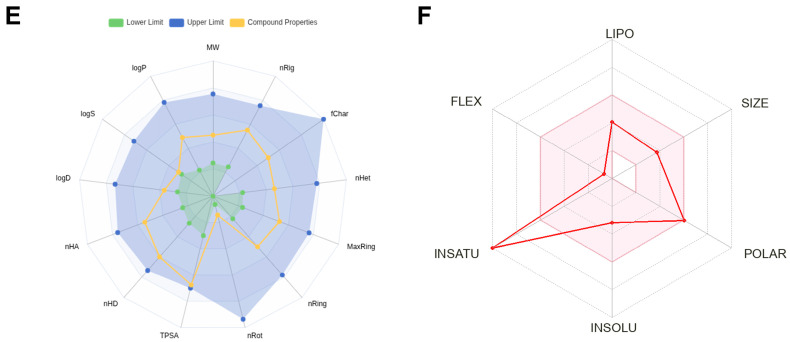
In silico analysis (**A**) Total binding energy (kcal/mol) of quercetin against *T. gondii* targets obtained by molecular docking simulations. (**B**) Binding affinity (kcal/mol) of quercetin against *T. gondii* targets. (**C**) Binding of quercetin in the active site of TgHGPRT. (**D**) Diagram of interactions showing hydrophobic and hydrogen-bond interactions between quercetin and amino acid residues involved in substrate binding and catalysis, including Glu146, Asp147, Asp150, Thr151, Gly152, and Asp206. (**E**) Radar plot showing predicted ADMET-related physicochemical parameters of quercetin compared with the recommended lower and upper limits. MW: molecular weight; logP: lipophilicity; logS: aqueous solubility; logD: distribution coefficient; nHA: number of hydrogen bond acceptors; nHD: number of hydrogen bond donors; TPSA: topological polar surface area; nRot: number of rotatable bonds; nRing: number of rings; MaxRing: maximum ring size; nHet: number of heteroatoms; TChar: total charge; nRig: number of rigid bonds. (**F**) Radar plot showing physicochemical properties., LIPO: lipophilicity; SIZE: molecular size; POLAR: polarity; INSOLU: solubility; FLEX: flexibility; INSATU: saturation. The pink area represents the optimal range for oral bioavailability.

To gain insights into the antiparasitic activity of quercetin, we evaluated the binding affinity against *T. gondii* targets through molecular docking simulations. Our results showed that quercetin exhibited the best binding to *T. gondii* hypoxanthine-guanine phosphoribosyltransferase (TgHGPRT) (Figure 7A,B). In the active site, quercetin formed hydrophobic and hydrogen-bond interactions with amino acid residues involved in the substrate binding and catalysis, including Glu146, Asp147, Asp150, Thr151, Gly152, and Asp206 (Figure 7C,D). The list of hydrogen-bonds is available in the Supplementary File S1 (Table S2). In addition, the prediction of the ADMET properties showed that quercetin presents good pharmacokinetics properties, such as gastrointestinal absorption, and drug-likeness (Figure 7E,F). The complete ADMET properties is available in the Supplementary File S2.

## 3. Discussion

Currently, conventional treatments for *T. gondii* present significant challenges due to their limitations, such as adverse effects, and still require further investigation [7,25]. Consequently, the study of alternative treatments has grown in recent decades [17,26,27], aiming to identify therapies with lower toxicity, greater specificity, and improved efficacy for the prevention or treatment of congenital toxoplasmosis [28]. Importantly, these limitations become even more critical in the context of pregnancy, in which the safety of medications must be ensured for both the mother and the fetus. In addition to concerns about toxicity, the possibility of parasite adaptation and reduced susceptibility to medications reinforces the need for the development of new therapeutic strategies.

In the case of quercetin, in vitro and in vivo studies demonstrated a cytoprotective and non-teratogenic effect during pregnancy [29]. Moreover, research has shown the beneficial effects of quercetin across various pathologies [30]. These include antitumor activities [31,32,33], anti-inflammatory action in autoimmune diseases (like rheumatoid arthritis) [34], and neurological activity in the treatment of Alzheimer’s [35]. It also exhibits preventive activity against cardiovascular diseases [36] and proven efficacy against *T. gondii* [21,23,37,38]. In this context, the present study investigated the treatment of congenital toxoplasmosis with quercetin. To provide a more scientific perspective, we combined in vitro, ex vivo, and in silico approaches, allowing for a broader assessment of the potential antiparasitic and host-modulating effects of quercetin.

Our results demonstrated that quercetin did not exhibit cytotoxicity at the tested concentrations, indicating therapeutic safety. In infected cells, quercetin exhibited a median inhibitory concentration (IC_50_) against *T. gondii* of 14.10 ± 2.83 μM, with a median cytotoxic concentration (CC_50_) > 100 μM, resulting in a selectivity index (SI) > 7.09. Moreover, *T. gondii* proliferation did not increase after the removal of the 24-h treatment, confirming the ability of quercetin to restrict parasite growth. Thus, these results showed that the damage caused was irreversible, as even after the removal of the treatment, the parasites did not resume proliferation. This was validated by TEM, where it was observed that the parasites exhibited morphological changes and intracellular vacuole-like structures. To validate the hypothesis that quercetin directly damages the parasites, the durability of the treatment was evaluated through a reinfection assay. In this setup, quercetin-exposed parasites demonstrated a reduced capacity to invade and proliferate in new host cells.

To investigate whether the action of quercetin also encompasses the host cell, pre-treatment assays were conducted on both the parasite and the cell. Pre-treatment of either host cells or parasites reduced parasite at most tested concentrations. Regarding invasion, all tested concentrations reduced invasion in host cell pre-treatment assays, while in parasite pre-treatment, only the 50 μM concentration was able to reduce invasion. This same pattern of invasion of pre-treated parasites was observed for the proliferation of pre-treated parasites, also being the only action seen from SP in this group of assays. These experiments demonstrate that quercetin exerts a dual action on both the parasite and the host cell. Furthermore, scanning electron microscopy corroborated these results, as the parasite pre-treated with quercetin exhibited structural damage, unlike SP, which maintained the common morphology of the parasite.

To better understand the biological basis underlying the observed irreversible antiparasitic effects, we then evaluated the pathways related to the metabolic interactions between host and parasite. Among these pathways, lipid metabolism represents a critical requirement for *T. gondii* replication, as the parasite depends on host-derived lipids for membrane biogenesis and intracellular growth [22,23]. Given that *T. gondii* is highly dependent on lipids, interference with the host’s lipid metabolism may represent an important strategy to restrict the growth of the parasite [22,39,40]. Thus, the ability of quercetin to inhibit enzymes involved in fatty acid synthesis corroborates our findings on lipid droplets. Accordingly, the reduction in lipid droplets observed here agrees with previous reports showing that quercetin can regulate cellular pathways involved in lipid synthesis, storage, and metabolic homeostasis [41,42]. Therefore, quercetin reduces lipid droplets, which serve as a source of nutrients for the parasite [43]. This reduction can create a metabolically unfavorable environment for parasite survival. In addition to metabolic requirements related to lipid availability, redox homeostasis is also essential for the survival of the parasite and the host cell response during infection, since host-derived oxidative stress is an important determinant of parasite adaptation and stage differentiation [19,44]. Moreover, lipid droplets play a regulatory role in oxidative stress. Therefore, we evaluated the antioxidant profile of quercetin in both uninfected and *T. gondii*-infected cells to verify whether its antioxidant capacity was maintained during infection [45].

In this context, biochemical markers of oxidative stress were also evaluated. The redox biochemical data support a model of context-dependent hormetic adaptation in which quercetin exerts distinct effects according to the cellular oxidative environment. Under non-infected conditions, quercetin (50 μM) behaved as a mild pro-oxidant. Autoxidation of its catechol moiety at physiological pH generates o-semiquinone radicals and low levels of O_2_•^−^ and H_2_O_2_ [46,47], which may account for the increase in intracellular ROS observed. This interpretation agrees with previous reports describing concentration-dependent redox effects of quercetin, including pro-oxidant activity at concentrations within the range used in this study [48]. Rather than causing overt oxidative damage, this moderate stimulus appears to have triggered an adaptive antioxidant response, evidenced by the expansion of the total thiol pool. Such a response is consistent with quercetin-induced Nrf2 activation and upregulation of γ-glutamylcysteine ligase, which may enhance intracellular thiol availability [38,49]. The reduction in SOD activity reported may likewise reflect an adaptive adjustment of the redox signaling network, as thiol-dependent antioxidant defenses become more prominent and O_2_•^−^ participates in signaling processes [50]. Importantly, unchanged AOPP levels indicate that the pro-oxidant stimulus remained below the threshold associated with oxidative injury, a hallmark of hormetic-like responses [48,51].

During *T. gondii* infection, the redox environment shifted substantially. Infection depleted host GSH and reduced SOD activity, findings consistent with previous reports describing parasite-mediated modulation of host antioxidant defenses [52,53]. The increase in catalase activity observed in infected cells may represent a compensatory response to elevated oxidative burden. In contrast, quercetin-treated infected cells showed restored GSH levels and increased total antioxidant capacity, suggesting reinforcement of antioxidant defenses. These findings are compatible with the activation of protective pathways, including Nrf2-dependent mechanisms previously associated with quercetin treatment [38,49]. The lower catalase activity observed in these cells may therefore reflect a reduced requirement for catalase-mediated H_2_O_2_ detoxification once GSH-dependent antioxidant pathways are restored.

The absence of significant differences in ROS levels among infected groups may result from a masking effect, whereby parasite-induced oxidative stress exceeds the relatively modest ROS contribution generated by quercetin, limiting the detection of subtle redox modulation [53]. Likewise, unchanged AOPP levels suggest that oxidative protein damage remained limited during the experimental period. Alternatively, oxidatively modified proteins may have been efficiently removed through proteasome-dependent quality-control mechanisms, which can operate over timescales extending beyond the treatment period used here [54,55].

Next, we evaluated a potential modulatory action of quercetin in cytokine production, and it was found that uninfected BeWo cells increased the secreted levels of IL-4, IL-6, and IL-8 in response to quercetin treatment. Previously, it was demonstrated, also in BeWo cells, the property of quercetin in promoting trophoblast syncytialization, wherein quercetin boosted cell fusion in BeWo cells through increasing expression of syncytial fusion markers and reducing mitochondrial ROS-dependent oxidative stress [56]. Interestingly, it is well known that syncytiotrophoblast cell population restricts *T. gondii* infection in the human placental environment [57,58]. Also, it is important to consider that appropriate levels of IL-4, IL-6, IL-8, and other immune mediators, at the maternal–fetal interface, are essential to ensure normal trophoblast function and pregnancy success [59,60,61], especially in the context of *T. gondii* infection [62]. Collectively, these reports and our findings suggest that the quercetin-induced increase in IL-4, IL-6, and IL-8 observed here may contribute, at least in part, to the establishment of a favorable microenvironment that promotes BeWo cell syncytialization, ultimately contributing to the control of *T. gondii* infection. This scenario is particularly plausible for IL-4 and IL-8 in the context of *T. gondii* infection, since quercetin treatment also increased the production of both cytokines by *T. gondii*-infected BeWo cells. Additionally, the quercetin-induced increase in IL-4 in BeWo cells, as was also observed for IL-6 and IL-8, may be a regulatory feedback response in an attempt to maintain IL-6 and IL-8 within physiological ranges, as excessive levels of IL-6 and IL-8 can exert inflammatory effects which may be detrimental to normal placental function and pregnancy [4,5,6]. Considering these observations, additional experiments in BeWo cells using quercetin in combination with a chemical inducer of trophoblast cell fusion, alongside chemical inhibitors or neutralizing antibodies for IL-4, IL-6, and IL-8, are needed to elucidate the contribution of trophoblast syncytialization and the involvement of these cytokines in the quercetin-mediated control of *T. gondii* infection. Subsequently, we corroborated our in vitro data by evaluating the ability of quercetin to control *T. gondii* infection in an ex vivo model using third-trimester human placental villous explants. As observed in the in vitro model, quercetin showed satisfactory cytotoxicity results, being non-toxic at all tested concentrations. In addition, the control of parasitic proliferation associated with the absence of cellular and structural damage, confirmed by the well-established and widely used MTT, LDH and histological analysis, may support the hypothesis that quercetin contributes to a balanced immune response, in which parasite control occurs without inducing significant tissue damage.

Finally, complementing the in vitro and ex vivo data, we conducted in silico assays to investigate the molecular targets of quercetin against *T. gondii* and to evaluate its drug-likeness properties. The investigation of the underlying mechanism of action for the development of effective therapeutic molecules can be assessed through molecular docking simulations [63,64]. In this study, we verified that quercetin binds to the active site of *T. gondii* hypoxanthine-guanine phosphoribosyltransferase (TgHGPRT) and forms hydrophobic and hydrogen bond interactions with amino acid residues involved in the substrate binding and catalysis. TgHGPRT plays a central role in the generation of purine nucleotides by transferring a ribosyl phosphate group from 5-phosphoribose 1-diphosphate (PRPP) to hypoxanthine, guanine or xanthine, producing IMP, GMP, and XMP, respectively [65,66], which could inhibit the parasite proliferation. Although the exact antiparasitic mechanism of action has not been fully addressed, our results provide valuable insights into the potential of quercetin for the investigation of effective molecules against *T. gondii* infection. Moreover, given that the knowledge of toxicity is essential for drug development, we assessed the ADMET properties in order to evaluate the drug-likeness of quercetin. Our results showed good pharmacological and physicochemical properties and do not violate the Lipinski, Pfizer or GSK rules (Supplementary File S2), which indicates good absorption, permeability and low toxicity [67,68]. However, despite these promising characteristics and the antiparasitic activity observed in the present study, the clinical application of quercetin remains challenging due to its relatively low bioavailability [23,69]. In this context, recent advances in drug delivery systems have demonstrated that nanoformulations [70], including polymeric nanoparticles, liposomes, and other nanocarrier-based approaches, can improve the stability, bioavailability, tissue targeting, and biological activity of flavonoids [71,72,73,74]. Therefore, future studies investigating quercetin-loaded nanocarriers may represent an important strategy to enhance its therapeutic potential against *T. gondii* infection, particularly in the context of congenital toxoplasmosis.

Based on our findings, quercetin appears to exert antiparasitic activity through multiple interconnected mechanisms involving both direct effects on the parasite and the modulation of host cell responses. These mechanisms have been previously described for flavonoids and include the regulation of redox signaling pathways, inflammatory mediators, cellular metabolism, and adaptive stress response mechanisms. In line with our data, it was demonstrated that Prunin could modulate this pathways, being an example of a bioflavonoid with antioxidant, anti-inflammatory and anti-cancer effects [75,76]. Within this context, the reduction in lipid droplets observed in the present study may help limit the availability of metabolic resources required for the development of intracellular parasites [43], while the modulation of antioxidant defenses and the restoration of redox balance may influence cellular homeostasis and host–parasite interactions [44]. Similarly, the cytokine profile induced by quercetin suggests an immunomodulatory effect that preserves tissue integrity and prevents excessive inflammatory responses [9,10,77,78].

In parallel, the ultrastructural changes observed by electron microscopy, together with the predicted interaction of quercetin with TgHGPRT, support the hypothesis that host-targeted effects may occur simultaneously with direct damage to the parasite. Collectively, these mechanisms may help explain the sustained inhibition of parasite proliferation and the absence of proliferative recovery following treatment withdrawal. Furthermore, the maintenance of cellular viability in the in vitro model and the preservation of tissue architecture in the ex vivo model suggest that quercetin contributes to placental homeostasis. Taken together, these findings reinforce the concept that the antiparasitic activity of quercetin likely results from the integration of metabolic, redox, immunological, and direct antiparasitic effects rather than from the modulation of a single molecular target.

## 4. Materials and Methods

### 4.1. Cell Culture and Parasite Maintenance

BeWo cell line, representative of villous trophoblasts, was obtained from the American Type Culture Collection (CCL-98TM, ATCC, Manassas, VA, USA). Cells were cultured in 75 cm^2^ flasks using RPMI 1640 medium (Cultilab, Campinas, SP, Brazil) supplemented with penicillin (100 U/mL) and streptomycin (100 μg/mL) (both from Sigma Chemical Co., St. Louis, MO, USA), as well as 10% heat-inactivated fetal bovine serum (FBS) (Cultilab, Campinas, SP, Brazil). Cultures were maintained at 37 °C in a humidified atmosphere containing 5% CO_2_.

*T. gondii* tachyzoites (RH strain, clone 2F1), expressing the β-galactosidase gene, were maintained through successive passages in BeWo cells. Parasites were cultured in RPMI 1640 medium supplemented with 2% FBS, penicillin (100 U/mL), and streptomycin (100 μg/mL), at 37 °C in a 5% CO_2_ atmosphere, as previously described [79]. All infection experiments were performed at a multiplicity of infection (MOI) of 1:1. Prior to each experiment, *T. gondii* tachyzoites were incubated with trypan blue, counted in a Neubauer chamber under an optical microscope, and assessed for viability and morphology. Only viable parasites, identified by negative trypan blue staining, clear cytoplasm, and the characteristic crescent-shaped morphology of tachyzoites, were used for subsequent experiments.

### 4.2. Quercetin

Quercetin powder (lot Q4951-10G; Merck–Sigma, Darmstadt, Germany) was commercially acquired and reconstituted in dimethyl sulfoxide (DMSO) to obtain a stock solution of 8272 μM. Prior to each experiment, quercetin was always freshly diluted in RPMI 1640 medium, resulting in a final DMSO concentration of <0.1%. This DMSO percentage is secure and non-toxic to BeWo cells, as previously reported [24].

### 4.3. Cell Viability Assay

To evaluate the cytotoxicity of quercetin in BeWo cells, cell viability was assessed using the colorimetric MTT assay (3-(4,5-dimethylthiazol-2-yl)-2,5-diphenyltetrazolium bromide) [80]. BeWo cells (3 × 10^4^ cells/100 µL/well) were seeded in 96-well plates, with each well containing 100 µL of RPMI 1640 medium supplemented with 10% FBS. After 18 h of incubation, when cells reached approximately 80–90% confluence, cells were treated with serial twofold dilutions of quercetin (100, 50, 25, 12.5, 6.25, 3.125, 1.56, and 0.78 µM) or just with culture medium for the negative control (untreated cells). These concentrations were chosen based on previous studies [37]. In parallel, cells were incubated with 0.097% DMSO, equivalent to the percentage used in the highest concentration tested (100 µM). After 24 h, cells were incubated with 10 µL of MTT solution (5 mg/mL) added to 90 µL of RPMI 1640 medium containing 10% FBS for 3 h at 37 °C and 5% CO_2_. Subsequently, 100 µL of a solution containing 10% sodium dodecyl sulfate (SDS) and 50% dimethylformamide (DMF) was added to solubilize the formazan crystals. Absorbance was measured at 570 nm using a microplate spectrophotometer (Multiskan™ FC Microplate Photometer, Thermo Fisher Scientific, Waltham, MA, USA). Results were expressed as percentage cell viability relative to the negative control, considered as 100%. Three independent assays were conducted in the cell viability assay. The median cytotoxic concentration (CC_50_) was obtained through extrapolation of the log-linear dose–response curve, focusing on the curve region crossing the 50% cytotoxicity threshold. The software used was GraphPad Prism (version 8.0.1).

### 4.4. T. gondii Intracellular Proliferation Assay

BeWo cells (3 × 10^4^ cells/100 µL/well) were seeded in 96-well culture plates to evaluate the *T. gondii* intracellular proliferation. After 18 h, cells were exposed to *T. gondii* tachyzoites (RH strain, clone 2F1) at a multiplicity of infection (MOI) of 1:1 (one parasite per cell) in RPMI 1640 medium supplemented with 2% FBS. After 3 h of infection, cells were washed with 1× PBS to remove non-internalized parasites [81]. Infected BeWo cells were then treated with serial twofold dilutions of quercetin (100, 50, 25, 12.5, 6.25, 3.125, 1.56, and 0.78 µM) or culture medium only (untreated cells) for 24 h at 37 °C in a 5% CO_2_ atmosphere. In addition, the gold-standard therapy combining sulfadiazine (800 μM, Sigma-Aldrich, St. Louis, MO, USA)) and pyrimethamine (32 μM, Vetranal^TM^, Sigma-Aldrich, St. Louis, MO, USA)) (SP) was employed at concentrations previously demonstrated to be effective against *T. gondii* while remaining non-toxic to BeWo cells [81]. *T. gondii* intracellular proliferation was quantified using a β-galactosidase colorimetric assay with the chlorophenol red-β-D-galactopyranoside substrate (CPRG; Roche Diagnostics, Mannheim, Germany), and absorbance was measured at 570 nm. Parasite quantification was determined by comparison with a standard curve generated from free tachyzoites (ranging from 1 × 10^6^ to 15.625 × 10^3^ total parasites), and results were expressed as a percentage of *T. gondii* intracellular proliferation, with the mean parasite number in untreated infected cells defined as 100% proliferation. The efficacy of each treatment condition was evaluated relative to this control. The half-maximal inhibitory concentration (IC_50_) against *T. gondii* was determined from dose–response inhibition curves (log[inhibitor] vs. normalized response, variable slope). The selectivity index (SI) was subsequently calculated as the ratio between the CC_50_ obtained for quercetin-treated BeWo cells and the IC_50_ determined for *T. gondii*-infected cells treated with quercetin [24]. Three independent assays were conducted in *T. gondii* intracellular proliferation assay.

### 4.5. Reversibility and Reinfection Assays

To evaluate whether the treatment remained effective after treatment removal, a reversibility assay was conducted as previously described [24]. BeWo cells (3 × 10^4^ cells/100 µL/well) were seeded in two 96-well culture plates. After 18 h, the cells were exposed to tachyzoites for 3 h at an MOI of 1:1, followed by washing with 1× PBS to remove non-invaded parasites. The experiment was conducted under two conditions: on the first plate, the infected cells were treated with quercetin (12.5, 25, and 50 µM), SP (800 µM and 32 µM, respectively), and only culture medium for 24 h; and after the treatment time, proliferation was measured. In parallel, and after the same treatment period, the cells in the second plate were washed and the medium was replaced with RPMI 1640 medium supplemented with 10% FBS, allowing the growth of the parasite to be monitored for an additional 24 h in the absence of treatment. In both conditions, the *T. gondii* intracellular proliferation was quantified using the β-galactosidase assay. Thus, the ability of the treatment to be reversible or not when the treatment is removed was analyzed.

To further investigate parasite recovery after drug exposure, a reinfection assay was employed. This assay was designed to determine whether parasites released from cells previously exposed to quercetin retain the ability to invade and replicate newly seeded host cells. In brief, BeWo cells were seeded at a density of 1 × 10^6^ cells per well in 6-well plates. After cell adhesion, cultures were infected at an MOI of 1:1 and incubated in RPMI 1640 medium for 3 h at 37 °C in a humidified atmosphere containing 5% CO_2_. The medium was then removed, and cells were washed with 1× PBS to eliminate non-invaded parasites. Subsequently, infected cells were treated for 24 h at 37 °C and 5% CO_2_ under the following conditions: only culture medium, SP (800 µM and 32 µM, respectively), and the selected concentration of quercetin (50 µM). After 24 h of treatment, parasites were obtained by serial passage through 21- and 26-gauge needles and counted using a Neubauer chamber. Tachyzoites recovered from treated BeWo cells were then used to infect new BeWo cells (3 × 10^4^ cells/100 µL/well) that were seeded in 96-well culture plates. Finally, a β-galactosidase assay was performed to quantify parasite levels at 3 h (invasion) and 24 h (proliferation) post-infection, and the results were expressed as the percentage of proliferation and invasion of the parasites relative to the negative control condition. Three independent assays were conducted in both reversibility and reinfection experiments.

### 4.6. Adhesion and Invasion Assays

The adhesion assay was performed as previously described [82,83], with minor modifications. BeWo cells (3 × 10^4^ cells/100 µL/well) were seeded in 96-well culture plates, and two experimental approaches were employed. In the first set of experiments, adhered BeWo cells were treated with quercetin (50 µM), SP (800 µM and 32 µM, respectively), and only culture medium for 24 h at 37 °C in a humidified atmosphere containing 5% CO_2_. After the treatments were removed, the cells were fixed with 4% paraformaldehyde for 30 min at room temperature, followed by washing with 1× PBS. Pretreated cells were then infected at an MOI of 1:1 and parasites were allowed to interact with previously fixed cells for 3 h at 37 °C and 5% CO_2_. In the second set of experiments, BeWo cells were fixed with 4% paraformaldehyde for 30 min prior to infection. Free tachyzoites used to infect these fixed cells were pretreated in microtubes for 1 h at 37 °C and 5% CO_2_ with quercetin (50 µM), SP (800 µM and 32 µM, respectively), or culture medium alone. Next, parasites were centrifuged at 402× *g* for 5 min, resuspended in a free culture medium, and used to infect at an MOI of 1:1 the fixed cells for 3 h under the same incubation conditions. In both approaches, parasite adhesion was quantified using a β-galactosidase assay, and results were expressed as the percentage of parasite adhesion relative to the control group treated with culture medium alone, which was defined as 100%.

In addition, an invasion assay was performed to evaluate both the ability of pretreated parasites to invade and proliferate and the capacity of pretreated host cells to prevent parasite invasion [84]. For the first experiment, BeWo cells (3 × 10^4^ cells/100 µL/well) were seeded in 96-well culture plates for 18 h at 37 °C and 5% CO_2_. Next, the parasites, at an MOI of 1:1, were pre-incubated for 1 h at 37 °C and 5% CO_2_ with quercetin (50 µM), SP (800 µM and 32 µM, respectively), and only culture medium. After the incubation period, the purified suspension of parasites, in treatment-free medium, was inserted into the plate previously seeded with BeWo cells, allowing the invasion of the pre-treated parasites for 3 h. In this same experimental setup, a second condition was evaluated, in which the suspension of pre-treated parasites for 1 h was added to plates previously seeded with BeWo and maintained for 3 h; however, after the infection period, the medium containing the treatment was removed and replaced with treatment-free medium for an additional 24 h, allowing for the analysis of the proliferation of pre-treated parasites. Moreover, a third experimental condition, conducted in independent plates, evaluated the pre-treatment of BeWo cells. For this experiment, BeWo cells (3 × 10^4^ cells/100 µL/well) were seeded in 96-well culture plates for 18 h at 37 °C and 5% CO_2_. Next, the cells were treated with only culture medium, SP (800 µM and 32 µM, respectively), and quercetin (50 µM) for 24 h. On the last day, *T. gondii* parasites were used at an MOI of 1:1 to infect these previously treated plates for 3 h, and the β-galactosidase assay was performed. Three independent assays were conducted in both adhesion and invasion experiments.

### 4.7. Scanning Electron Microscopy (SEM)

Scanning electron microscopy (SEM) was performed to analyze the direct effects of quercetin on *T. gondii*. For this assay, 1 × 10^7^ parasites/1000 µL (*T. gondii* RH strain, clone 2F1) were treated with quercetin (50 µM), SP (800 µM and 32 µM, respectively), and only culture medium for 1 h at 37 °C in a humidified atmosphere containing 5% CO_2_. After treatment, parasites were centrifuged and washed with potassium cacodylate buffer, followed by fixation with Karnovsky’s solution (2% glutaraldehyde and 2% paraformaldehyde) for 3 h. After fixation, samples were washed again with potassium cacodylate buffer and post-fixed with 1% osmium tetroxide (OsO_4_) for 1 h. Subsequently, concentrated parasites were smeared onto circular glass coverslips (13 mm in diameter) and allowed to dry for 18 h at room temperature. Next, samples were dehydrated through a graded ethanol series (50%, 70%, 80%, 90%, 95%, and 100%). Finally, samples were coated with a thin layer of gold and examined using a scanning electron microscope (Tescan Vega-3 LMU, Brno, Czech Republic) [85].

### 4.8. Transmission Electron Microscopy (TEM)

Transmission electron microscopy (TEM) was performed to analyze the ultrastructure of intracellular *T. gondii* tachyzoites and host cell morphology. For this assay, BeWo cells were cultured and infected with *T. gondii* tachyzoites (RH strain, clone 2F1) at an MOI 1:1 for 3 h. Subsequently, cells were treated with quercetin (50 µM) and only culture medium for 24 h at 37 °C in a humidified atmosphere containing 5% CO_2_. After treatment, cells were harvested and fixed with Karnovsky’s solution containing 2% paraformaldehyde and 2% glutaraldehyde in 0.1 M sodium cacodylate buffer (pH 7.4) for 24 h. Samples were then washed three times with PBS and post-fixed with 1% osmium tetroxide (OsO_4_) in cacodylate buffer for 1 h. Two independent assays with two replicates each were conducted in this analysis. Subsequently, samples were processed as previously described and examined using transmission electron microscopes HT-7700 (Hitachi, Tokyo, Japan) and EM900 (Carl Zeiss, Oberkochen, Germany).

### 4.9. Lipid Droplets (LDs) Staining

Lipid droplets (LDs) accumulation in quercetin-treated BeWo cells was evaluated. Briefly, BeWo cells (3 × 10^4^ cells/100 µL/well) were seeded in black 96-well culture plates with clear bottoms (Costar REF# 3603, New York, NY, USA). After 18 h, cells were infected or not at a MOI of 1:1 for 3 h at 37 °C in a humidified atmosphere containing 5% CO_2_. Cells were then washed with 1× PBS to remove non-internalized parasites and subsequently treated or not with quercetin (50 µM), SP (800 µM and 32 µM, respectively), and only culture medium for 24 h under the same incubation conditions. After treatments were removed, the cells were fixed with 4% formaldehyde, washed twice with 1× PBS, and stained with Nile Red (Sigma Chemical Co., St. Louis, MO, USA; cat. no. 72485) (diluted 1:1000 in 1× PBS) to label LDs according to previously described protocols [86,87]. Following staining, excess dye was removed, and cells were washed with 1× PBS prior to fluorescence measurement. Nile Red fluorescence was measured using a multiwell scanning spectrophotometer (VersaMax, Molecular Devices, San Jose, CA, USA) at an excitation wavelength of 520 nm and emission between 580 and 640 nm. Three independent assays were conducted in the lipid droplet assay. The images were captured using a fluorescence microscope (EVOS fl, Thermo Fisher) and, after analysis, representative images were selected for illustration of the phenomenon.

### 4.10. Cytokine Quantification

Levels of the cytokines IL-4, IL-6, IL-8, MIF, and TNF-α were quantified in culture supernatants from BeWo cells, either infected or not with *T. gondii*. The experimental conditions included treatment with quercetin (50 µM), SP (800 µM and 32 µM, respectively), and only culture medium. Cytokine concentrations were measured using enzyme-linked immunosorbent assay (ELISA) kits according to the manufacturers’ instructions (BD Biosciences, San Diego, CA, USA; R&D Systems, Minneapolis, MN, USA), following previously described methodologies [88,89]. Cytokine levels were expressed in pg/mL, and the detection limits for each cytokine were determined based on the standard curves: IL-6 (4.7 pg/mL), IL-8 (6.25 pg/mL), MIF (93.8 pg/mL), IL-4 and TNF-α (both 7.8 pg/mL). One assay was conducted using three supernatants from independent experiments.

### 4.11. Evaluation of Oxidative Stress Biomarkers

Oxidative stress assays were performed to analyze the effects of quercetin on BeWo cells under infected and non-infected conditions. For this assay, BeWo cells (1 × 10^6^ cells/2000 µL/well) were seeded in 6-well culture plates. After cell adhesion, part of the cultures was infected with *T. gondii* (RH strain, clone 2F1) at an MOI of 1:1, while the remaining cultures were maintained as non-infected controls. Cells were treated with quercetin (50 µM), SP (800 µM and 32 µM, respectively), and only culture medium for 24 h at 37 °C in a humidified atmosphere containing 5% CO_2_. After treatment, the culture medium was removed, and the cells were washed with PBS. Subsequently, 1000 µL of PBS containing KCl (140 mM) was added to each well, and cells were mechanically detached using a cell scraper and transferred to 1.5 mL microtubes. The cell suspensions were homogenized and subjected to three freeze–thaw cycles for cryolysis. After cryolysis, samples were kept on ice and used for the antioxidant assays described below. At the end of each assay below, the total protein concentration was determined by the Bradford method and all oxidative stress biomarkers were normalized to the total protein content [90]. In these experiments each biomarker had three replicates.

#### 4.11.1. Reactive Oxygen Species (ROS) Content

ROS levels were evaluated by incubating the cell lysates with dichlorodihydrofluorescein diacetate (10 µM, prepared in ethanol) in the presence of 5 mM Tris–HCl buffer (pH 7.4). After a 3 min incubation period, fluorescence emission was measured at 530 nm following excitation at 474 nm in a 96-well microplate LS 55 (Perkin-Elmer Waltham, MA, USA). ROS levels were expressed as relative luminescence units [90].

#### 4.11.2. Advanced Oxidation Protein Products (AOPP) Content

AOPP levels were determined by incubating the cell lysates with 0.2 M citric acid prepared in phosphate buffer (pH 7.4) in a 96-well microplate. Following a 2 min agitation period on a microplate shaker, absorbance was recorded at 340 nm (Perkin-Elmer LS 55), with citric acid serving as the solvent blank. Quantification was performed based on a calibration curve generated with chloramine-T in the presence of 1.16 M potassium iodide (KI) as the reference standard. AOPP concentrations were expressed as micromolar (μM) chloramine-T equivalents [91].

#### 4.11.3. Sulfhydryl Group Content

Free sulfhydryl (thiol) group levels were determined according to the method described by Ellman (1959) [92], using 5,5′-dithiobis (2-nitrobenzoic acid) (DTNB) as the chromogenic reagent. Cell lysates were incubated with DTNB solution (10 mM, prepared in potassium phosphate buffer, pH 8.0) for 30 min. Absorbance was measured at 412 nm (Molecular Devices) and thiol concentrations were calculated based on a molar extinction coefficient of 14,150 M^−1^·cm^−1^.

#### 4.11.4. Superoxide Dismutase (SOD) Activity

SOD activity was determined according to the method described by Marklund and Marklund [93], with minor modifications. This assay is based on the inhibition of pyrogallol auto-oxidation mediated by superoxide radicals. The cell lysates were incubated with catalase (80 U/mL) and pyrogallol (24 mM) prepared in 50 mM Tris–HCl buffer supplemented with 1 mM EDTA (pH 8.2). Kinetic measurements were performed in 96-well microplates by monitoring absorbance at 420 nm over a 10 min interval (Molecular Devices). SOD activity was quantified from a calibration curve constructed with purified SOD as the reference standard [90].

#### 4.11.5. Catalase (CAT) Activity

CAT activity was determined according to the method described by Aebi [94], with minor modifications. This assay is based on the decomposition of hydrogen peroxide (H_2_O_2_). The cell lysates were incubated with an H_2_O_2_ solution (0.2% prepared in 10 mM phosphate buffer, pH 7.0). Enzymatic activity was monitored kinetically in 96-well microplates by measuring the decrease in absorbance at 240 nm over a 10 min period (Perkin-Elmer LS 55).

#### 4.11.6. Reduced Glutathione (GSH) Content

GSH levels were determined in cell lysates following protein precipitation with metaphosphoric acid (1:1, *v*/*v*). The samples were centrifuged at 7000× *g* for 10 min, and the resulting supernatants were subsequently mixed with sodium phosphate buffer and o-phthalaldehyde (1 mg/mL, prepared in methanol). Fluorescence was measured in 96-well microplates at excitation and emission wavelengths of 350 nm and 420 nm, respectively (Perkin-Elmer LS 55). GSH concentrations were calculated based on a calibration curve generated with reduced glutathione as the standard reference [95].

#### 4.11.7. Total Antioxidant Capacity

Total antioxidant capacity was determined using the ferric reducing antioxidant power (FRAP) assay, adapted from the method described by Benzie and Strain [96]. Briefly, the FRAP reagent was prepared by mixing 0.3 M acetate buffer (pH 3.6), 10 mM 2,4,6-tris(2-pyridyl)-s-triazine (TPTZ) in acidic solution, and 20 mM ferric chloride at a 10:1:1 ratio. Cell lysates were incubated with the FRAP reagent at 37 °C for 6 min, and absorbance was subsequently measured at 593 nm in 96-well microplates (Molecular Devices). Antioxidant capacity was quantified based on a standard curve constructed with 6-hydroxy-2,5,7,8-tetramethylchroman-2-carboxylic acid (Trolox) and expressed as Trolox equivalents [90].

### 4.12. Collection of Human Villous Explants

To support the findings on the effects of quercetin, an ex vivo model using placental explants was employed. Third-trimester placentas (36–40 weeks of gestation) were obtained from pregnant women aged 18–45 years undergoing elective cesarean deliveries at the Clinical Hospital of the Universidade Federal de Uberlândia (UFU), with no reported comorbidities. Conditions such as diabetes, hypertension, preeclampsia, cardiovascular diseases, toxoplasmosis, chagas disease, or any other condition that could interfere with the results were considered exclusion criteria. For the preparation of human chorionic villous explants (HCVE), placentas were washed with sterile 1× PBS to remove excess blood and debris. Placental cotyledons were then dissected, and chorionic villi were obtained according to an established protocol. Selected villi (~10 mm^3^) were individually placed into wells of a 96-well plate containing 200 µL of RPMI 1640 medium supplemented with 10% FBS, penicillin, and streptomycin. Samples were incubated at 37 °C in a humidified atmosphere with 5% CO_2_ for subsequent assays [97,98,99]. To account for donor-to-donor variability, experiments were performed using placentas from 3 independent donors. All procedures were approved by the Human Research Ethics Committee of the Universidade Federal de Uberlândia (CEP/UFU; approval number 7.407.162), and written informed consent was obtained from all participating pregnant women.

### 4.13. Viability Assays of Human Villous Explants

To evaluate the viability of placental villi following quercetin treatment, lactate dehydrogenase (LDH) release and MTT assays were performed, as previously described [80,99]. Collected placental villi were cultured for 24 h at 37 °C in a humidified atmosphere containing 5% CO_2_. Subsequently, villi were treated or not with quercetin (50 µM), SP (600 µM and 804 µM, respectively), and only culture medium for an additional 24 h. Also, the DMSO vehicle was tested at the highest concentration percentage (0.097%) corresponding to 100 µM. After treatment, culture supernatants were collected for LDH quantification according to the manufacturer’s instructions. For viability assessment by the MTT assay, after removal of the supernatants, 200 µL of MTT solution [180 µL of culture medium plus 20 µL of MTT (5 mg/mL)] was added and incubated for 4 h at 37 °C and 5% CO_2_. Formazan crystals were then solubilized by the addition of 10% SDS and 50% DMF, and after 24 h (only the supernatant, the villi were removed), absorbance was measured at 570 nm using a microplate reader (Multiskan™ FC Microplate Photometer, Thermo Fisher Scientific, Waltham, MA, USA). In addition, villous tissues treated with quercetin (50 µM), SP (600 µM and 804 µM, respectively), or culture medium alone were subjected to hematoxylin and eosin staining and examined under a light microscope (BX40; Olympus, Tokyo, Japan) [99]. The assessment of viability through MTT assay, LDH release and histological analysis represents a widely accepted standard in placental explant literature to confirm tissue integrity and viability [24,100,101]. Three independent assays were conducted with eight replicates in viability of human villous explants.

### 4.14. T. gondii Intracellular Proliferation in Human Placental Explants

Infection assays in placental explants were performed to analyze the effects of quercetin on *T. gondii* intracellular proliferation. For this assay, placental explants were placed in 96-well microplates and cultured for 24 h at 37 °C in a humidified atmosphere containing 5% CO_2_. After this period, explants were infected with *T. gondii* tachyzoites (RH strain, clone 2F1) (1 × 10^6^ parasites/200 µL/well) and incubated for an additional 24 h under the same conditions. Subsequently, explants were treated with quercetin (50 µM), SP (600 µM and 804 µM, respectively), and only culture medium for 24 h at 37 °C in a humidified atmosphere containing 5% CO_2_. After treatment, culture supernatants were collected and stored at −80 °C for subsequent determination of total protein content using Bradford reagent (Sigma) and analysis of *T. gondii* intracellular proliferation by β-galactosidase assay, as previously described [98,99]. Three independent assays were conducted with eight replicates to *T. gondii* intracellular proliferation.

### 4.15. In Silico Analysis

In order to gain insights into the antiprotozoal mechanism of quercetin, we performed molecular docking simulations against *T. gondii* targets. The targets (Supplementary File S1 (Table S1)) structures were obtained from AlphaFold-DB [102] retrieved from UniProt [103],while the structure of quercetin was obtained from PubChem [104], under accession code 5280343. The molecular docking simulations were performed using Dockthor v.2 [105], with a grid box of 20 Å for atom selection from the annotated binding sites present in UniProt (Supplementary File S1), and RMSD of 2.0 Å as clustering criterion. The docking was ranked by the total energy calculated from the MMFF94S force field [105]. The results were visualized using matplotlib (version 3.11.0) [106], pandas (version 3.0.3) [107], and seaborn (version 0.113.2) [108] Python (version 3.14.5) (https://www.python.org/) packages. The interactions were analyzed using Discovery Studio Visualizer v. 24.1.0 (BIOVIA, Dassault Systèmes, San Diego, CA, USA). In addition, to evaluate the pharmacokinetic and safety of quercetin, we predicted the ADMET (absorption, distribution, metabolism, excretion, and toxicity) properties using ADMETLab v.3.0 [67] and SwissADME [68].

### 4.16. Statistical Analysis

Statistical analyses and graphical representations were generated using GraphPad Prism software (version 8.0.1). Data are presented as mean ± standard error of the mean (SEM). Parametric data were analyzed using one-way analysis of variance (ANOVA), followed by Dunnett’s post hoc test for multiple comparisons. Nonparametric data were analyzed using the Kruskal–Wallis test. A *p*-value < 0.05 was considered statistically significant, indicating meaningful differences among treated groups and between treated and control groups.

## 5. Conclusions

In conclusion, our findings demonstrated that quercetin exhibits anti-*T. gondii* activity in the tested models, promoting an irreversible compromise of the parasite’s viability while maintaining the integrity of the host cells. Quercetin exerts a dual action by directly affecting the structure and proliferation of the parasite and by modulating the metabolic, redox, and immunological pathways of the host cell. The reduction in the availability of lipid droplets, the modulation of antioxidant defenses, and the regulation of cytokine production suggest that quercetin creates a metabolically and immunologically unfavorable environment for the survival of the parasite and protective for the host cell. Furthermore, the potential of quercetin is supported by its binding to the active site of the TgHGPRT enzyme and by the findings on favorable pharmacological and pharmacokinetic properties, conducted in our in silico analysis. Finally, when discussing the limitations of this study and future perspectives, it is important to emphasize the need for research with a longer action period of quercetin, as well as trials using new models, such as in vivo, for a deeper understanding of quercetin’s anti-*T. gondii* capacity.

## Data Availability

The original contributions presented in this study are included in the article/Supplementary Materials. Further inquiries can be directed to the corresponding author.

## Supplementary Materials

The following supporting information can be downloaded at: https://www.mdpi.com/article/10.3390/ijms27136054/s1.
